# Differences in spatial distributions of iron supplementation use among pregnant women and associated factors in Ethiopia: evidence from the 2011 national population based survey

**DOI:** 10.1186/s12884-016-1210-7

**Published:** 2017-01-14

**Authors:** Demewoz Haile, Lianna Tabar, Yihunie Lakew

**Affiliations:** 1 School of Public Health, College of Health Sciences, Addis Ababa University, Addis Ababa, Ethiopia; 2 WEEMA International, Brookline, MA USA; 3 Ethiopian Public Health Association, Addis Ababa, Ethiopia

## Abstract

**Background:**

Iron supplementation during pregnancy prevents against low birth weight, incidence of prematurity and postpartum hemorrhage. However, the coverage of iron supplementation is still low in Ethiopia. This study aimed to investigate the spatial variations and associated factors of iron supplementation during pregnancy using the 2011 national demographic and health survey data.

**Methods:**

This study used secondary data from the 2011 Ethiopian demographic and health survey. The survey was cross sectional and used a multistage cluster sampling procedure. A logistic regression statistical model using adjusted odds ratio (AOR) and 95% confidence interval (CI) was used to identify the associated factors. Getis-Ord G-statistic was used to identify high and low hotspot areas of iron tablet supplementation during pregnancy.

**Results:**

The coverage of iron tablet supplementation was 17.1% [95%CI: (16.3–17.9)] with the highest coverage of 38.9% [95%CI: (32.4--46.1)] in Addis Ababa followed by Tigray regional state with 33.8% [95%CI: (29.9–38.00)]. The lowest coverage was found in Oromiya regional state at 11.9% [95%CI: (10.7–13.0)]. Multivariable analysis showed that mothers who were aware of the Community Conversation Program had 20% [AOR = 1.2; 95% CI: (1.04–1.4)] higher odds of taking iron tablets. The odds of taking iron tablets was 2.9 times [AOR = 2.9; 95% CI: (2.3–3.7)] higher among those who took deworming tablets. Those mothers who attended the minimum four antenatal visits recommended by WHO were 3.9 times [AOR = 3.9; 95% CI: (3.3–4.6)] more likely and those mothers in the age group 31–49 years were 2.9 times [AOR = 2.9; 95% CI: (1.1–7.4)] more likely to use iron tablets as compared to those mothers who did not attend antenatal care and mothers in the age group less than 20 years. Mothers having a family size of 10 and above had 32% [AOR = 0.68; 95% CI: (0.49–0.97)] lower odds of taking iron tablets during pregnancy. The spatial analysis found that only northern, central and eastern parts of Ethiopia were identified as hotspots of iron supplementation.

**Conclusion:**

Iron supplementation use was not equally distributed in Ethiopia, with relatively higher prevalence in Tigray, Addis Ababa and Harari regional states. Attention should be given to younger age mothers, mothers with large family size and mothers who reside in areas with low coverage of iron tablet distribution. Promotion of antenatal care services based on the WHO standard can be used as an intervention for improving iron supplementation during pregnancy.

## Background

Iron is essential for blood production and a component of hemoglobin for carrying oxygen in the blood. Iron deficiency is one of the most preventable nutritional deficiency diseases among women worldwide and particularly prevalent during pregnancy [1]. During pregnancy, the intake of iron is recommended to be 27 mg per day which is 50% higher than required for non-pregnant women [2]. These iron requirement during pregnancy are extraordinarily high and cannot be fulfilled by dietary interventions alone [3, 4]. The low bioavailability of iron combined with high iron requirement during pregnancy especially in developing countries question extra source of iron such as from supplement [5].

As a response to this demand, routine supplementation of iron with folic acid is recommended by WHO for all pregnant women [5]. Particularly where anemia prevalence is high, it is recommended for iron supplementation to continue into the postpartum period to enable women to acquire adequate stores of iron [6, 7]. Iron supplementation during pregnancy prevents low birth weight [6–8]. Most importantly, iron supplementation during the first trimester of pregnancy among poor women improves birth weight and lowers the incidence of prematurity [9]. Iron supplementation is also associated with reducing the risk of postpartum hemorrhage [10]. As a result, daily oral iron and folic acid supplementation is recommended as part of antenatal care to reduce the risk of low birth weight, maternal anemia and iron deficiency [5]. The current recommendation is a 6 month regimen of a daily supplement containing 60 mg of elemental iron along with 400 mcg of folic acid [11].

In Ethiopia, the coverage of iron supplementation during pregnancy is still low and has not fulfilled the WHO standard recommendations. This study aimed to investigate the differences in spatial distributions of iron supplementation and associated factors among pregnant women in Ethiopia using the 2011 demographic and health survey data.

## Methods

### Study setting

The 2011 Ethiopian Demographic and Health Survey (EDHS) was conducted in nine regional states of Ethiopia namely; Tigray, Afar, Amhara, Oromia, Somali, Benishangul-Gumuz, Southern Nations Nationalities and Peoples (SNNP), Gambella and Harari and two city Administrations (Addis Ababa and Dire Dawa). Ethiopia is one of the sub-Saharan countries found in the Horn of Africa with 73.5 million with a populations of according to 2007 national housing and population census [12].

### Data type and study design

Data for this analysis was taken from the 2011 Ethiopian Demographic and Health Survey (EDHS 2011). The sample for the survey was designed to represent national, urban–rural, and regional estimates of health and demographic outcomes. The 2011 EDHS samples were selected using a stratified, two-stage cluster sampling design. In the first stage, 624 clusters of census enumeration areas (EAs), 187 in urban and 437 in rural areas were included in the survey. In the second stage, a complete listing of households was carried out in each of the 624 selected EAs from September 2010 through January 2011. Sketch maps were drawn for each of the clusters, and all conventional households were listed. A representative sample of 17,817 households was selected for the 2011 EDHS. Subsequently a total of 16,515 women in the age group 15–49 years who were usual residents or who slept in the selected households the night before the survey were eligible and interviewed for the survey. Among those women interviewed, 7764 were pregnant mothers who had pregnancy in the preceding 5 years [13, 14].

For this analysis, information on a wide-range of potential independent variables including socio-demographic, economic variables and health service related factors and iron supplementation during pregnancy as a dependent variable were extracted from the DHS data warehouse for 7764 pregnant mothers. Educational status of women and partner, birth interval, family size, age of the women, parity, occupation (working vs not working), residence (urban vs rural) and region where the respondent reside were the socio-demographic variables extracted from the data set for this study. Wealth index was used to measure the socio-economic status, to indicate inequalities in household characteristics. The index constructed serves as an indicator of level of wealth that is consistent with expenditure and income measures. Wealth index was constructed using household asset data via principal components analysis to categorize individuals into wealth quintile (poorest, poorer, medium, richer and richest). Variables included in the construction of the wealth index were ownership of selected household assets, size of agricultural land, quantity of livestock and materials used for house construction [14]. Health service related factors such as antenatal care attendance for the indexed pregnancy, anemia status (anemic vs non anemic), deworming tablet intake during the indexed pregnancy (yes or no), mass media exposure (exposure to mass media (indexed from television, newspaper and radio), awareness of Community Conversation (CC) program were extracted. Community Conversation is a health information delivery program which is implemented in rural communities of Ethiopia to improve the awareness of the community on different topics such as ANC, pregnancy and nutrition. The community members discuss each other on different issues of health with guidance from the community health workers (health extension worker). The discussion sometimes might be moderated by health development army, who serve as an assistant for the community health worker. The iron supplementation was collected from self-reported by showing the iron tablet by asking “During this pregnancy, were you given or did you buy any iron tablets? In this study, we used ever use of iron tablet as a dependent variable.

### Spatial analysis

Spatial analysis was applied to detect geographic variation among EDHS clusters through the application of Geographic Information System (GIS), an ArcGIS software of version 10.0 produced by ESRI, Redlands, CA, USA. The GPS points were downloaded with permission from Measure DHS and merged with the coverage of iron supplementation in each DHS study clusters. The coverage of iron tablet supplementation during pregnancy was exported into ArcGIS to visualize clusters of hot and low spots. Spatial heterogeneity of significant high coverage or low coverage of iron tablet supplementation were computed for each cluster using the Getis-Ord G-statistic tool in ArcGIS. To determine the significance of these statistics, Z-scores and P-value were used. A z-score near zero indicates no apparent clustering within the study area. A positive z-score with P-value of <0.05 indicates clustering of statistically high hotspots of iron supplementation, whereas a negative z-score with p-value of <0.05 indicates clustering of statistically low spots of iron supplementation. Maps to show the distribution and variations of iron supplementation throughout the country were constructed.

### Statistical analysis

Descriptive statistics including prevalence and frequency distributions were used to determine the level of iron supplementation. Bivariate analysis was used to show the association between socio-demographic characteristics and iron tablet use. Variables that were determined statistically significant at p-value <0.25 during bivariate analysis were considered for adjustment in the level of multivariable logistic regression model [15, 16]. This cutoff point prevented removing variables that would potentially have an effect during multivariable analysis. A stepwise approach was used to assess the iteration of variables and to control potential confounders [17]. We had checked the model by entering different variables step by step. The model with high value of iteration was selected as our final model in the multivariable logistic regression. In the multivariable model, odds ratio with 95% CI was used. A multi-collinearity test was done and as a result no variables had collinearity with variance inflation factors (VIF) of greater than 10 [18]. Sample weights were applied in order to compensate for the unequal probability of selection between the strata that were geographically defined as well as for non-responses. A detailed explanation of the weighting procedure can be found in the 2011 EDHS [19]. The “svy” command in STATA version 11 (Stata Corporation, College Station, TX, USA) was used to weight the survey data.

## Results

**Fig. 1:**
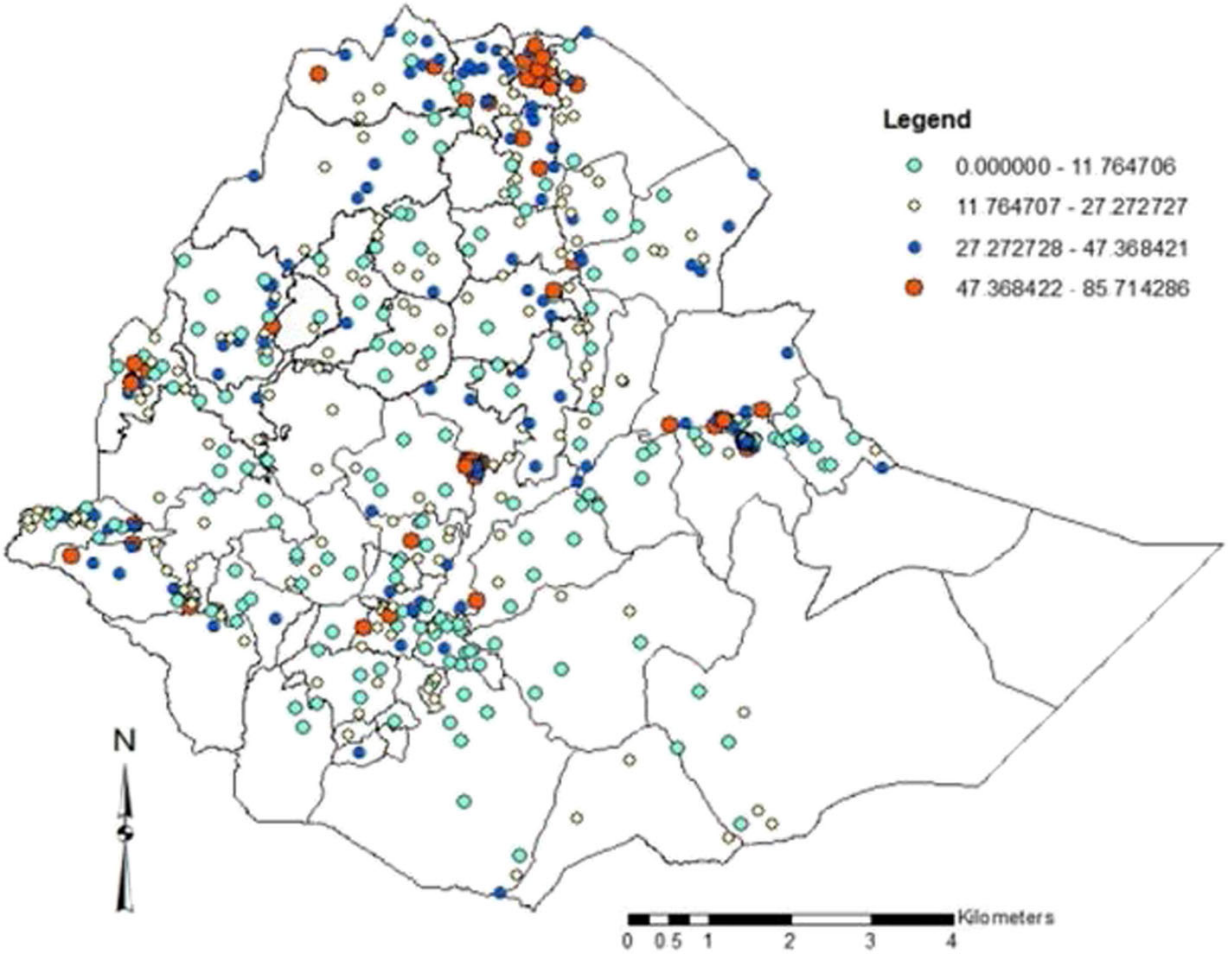
Iron intake during pregnancy spatial distribution in Ethiopia, 2011

The overall coverage of iron supplementation was 17.1% with [95%CI: (16.3–17.9)]. The highest coverage of iron tablet intake during pregnancy was found in Addis Ababa at 38.9% [95% CI: (32.4–46.1)], followed by Tigray regional state with 33.8% [95% CI (29.9–37.9)]. As shown on Fig. 1, the coverage of iron supplementation reaches up to 46 to 86% in certain geographic clusters particularly in the northern, central, eastern and western parts of the country. There are also some clusters with coverage of iron supplementation during pregnancy from 27.3 to 43.4% in few clusters all over the country. There were also a few numbers of clusters who had iron supplementation coverage from 11.8 to 27.3%. There were no EDHS clusters (enumeration areas) included in the peripheral eastern parts of the country, particularly in Somalia region, unable to estimate the coverage.

**Fig. 2:**
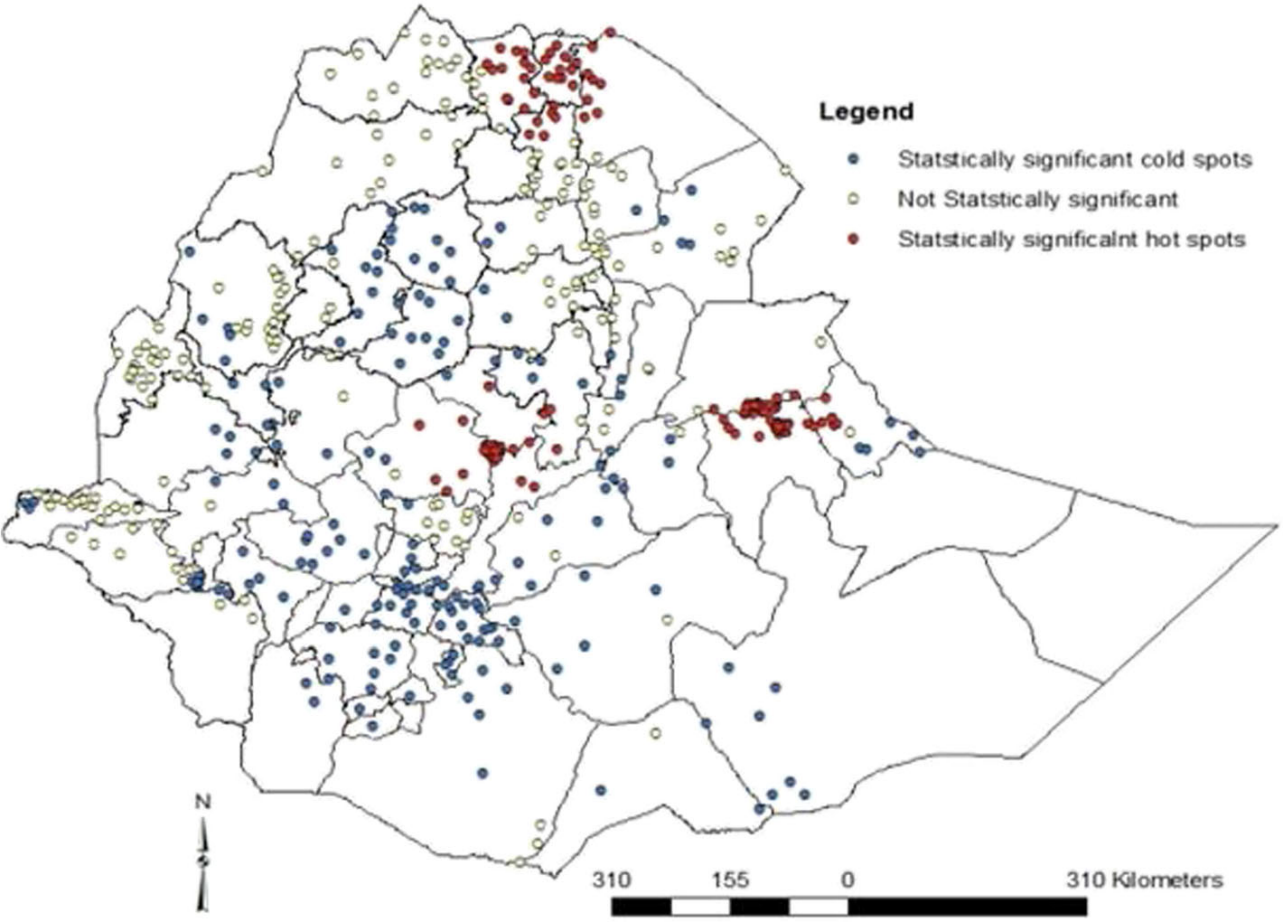
Map to display hot and cold spot clusters of iron supplementation during pregnancy in Ethiopia, 2011

The Getis-Ord G-statistic showed that iron supplementation during pregnancy was not uniform in Ethiopia. Figure 2 showed the spatial variation of iron supplementation during pregnancy at the cluster level (lower level). The analysis at the cluster level shows that statistically significant high hot-spots of iron tablet intake during pregnancy were found in Tigray region, northern parts of the country, Addis Ababa, Central Ethiopia, and Harar and Dire Dawa, Eastern Ethiopia whereas statistically significant low spots of iron intake during pregnancy were found in most part of the country i.e. the Northern Ethiopia (Amhara region, and few clusters of Benshangul-Gumuz and Affar region), most parts of Oromiya region, South nation, nationalities and people Region, South west Ethiopia (few clusters of Gambella region) (Fig. 2).

The lowest coverage was found in Oromiya region at 11.9% [95% CI: (10.7–13.0)]. The coverage of iron intake in urban settings was found to be 27.3% [95% CI: (24.8–29.9)]. About 23% [95% CI: (21.9–25.0)] of pregnant mothers from the rich wealth tertile had received iron tablet supplementation during their last pregnancy. Similarly the coverage of iron supplementation was 35.6% [95% CI: (30.8–40.66)] and 28.0% [95% CI: (24.8–31.3)] among mothers who had at least secondary education and mothers who had husband with a secondary education level and above, respectively (Table 1).

**Table 1:** Iron supplementation during pregnancy by background characteristics in Ethiopia, 2011

Background characteristics	Weighted frequency and percentage		Coverage of iron supplementation with 95%CI
	Frequency	Percentage	
Region			
Tigray	526	8.1	33.8 (29.9–38.00)
Afar	78	1.2	23.4 (14.9–33.8)
Amhara	1984	30.5	19.1 (17.4–20.8)
Oromia	3109	47.8	11.9 (10.7–13.0)
Somali	197	3.0	20.0 (14.7–25.8)
Benshangul-gumz	92	1.4	23.4 (15.2–31.8)
SNNPR	246	3.8	15.1 (13.4–16.9)
Gambella	31	0.5	27.5 (12.8–43.2)
Harari	19	0.3	31.6 (13.9–54.5)
Addis Ababa	192	3.0	38.9 (32.4–46.1)
Dire Dawa	26	0.4	28.2 (12.6–46.1)
Residence			
Urban	1183	15.0	27.3 (24.8–29.9)
Rural	6,699	85.0	15.3 (14.4–16.2)
Wealth index			
Poor	3420	43.4	13.0 (12.0–14.2)
Middle	1622	20.6	15.2 (13.5–17.0)
Rich	2840	36.0	23.1 (21.9–25.0)
Current occupation status			
Not working	3499	44.4	15.6 (14.4–16.8)
Working^*^	4383	55.6	18.3 (17.2–19.4)
Religion			
Christian	5157	65.4	17.9 (16.9–19.0)
Muslim	2552	32.4	15.9 (14.5–17.4)
Others	173	2.2	9.2 (5.6–14.3)
Community conversation			
Not heard	878	30.4	15.0 (14.1–15.9)
Heard	2008	69.6	23.3 (21.5–25.2)
Mother education			
None	5249	66.6	14.9 (13.9–15.9)
Primary	2266	28.6	19.2 (17.6–20.8)
Secondary and higher	368	4.7	35.6 (30.8–40.6)
Husband education			
None	3847	49.7	14.1 (13.0–15.2)
Primary	3171	40.9	17.9 (16.6–19.3)
Secondary and higher	729	9.4	28.0 (24.8–31.3)
ANC use			
0–3 times	6350	80.6	12.0 (11.2–12.8)
4+ times	1553	19.4	38.1 (35.6–40.6)
Anemia status			
Anemic	1429	18.8	14.5 (12.7–16.4)
Not anemic	6181	81.2	18.0 (17.1–19.0)
Parity			
1–4	4849	61.5	17.1 (16.1–18.2)
5–9	2703	34.3	17.1 (15.8–18.6)
10+	330	4.2	15.8 (12.1–20.0)
Taking Deworming tablet			
No	7393	94.4	15.6 (14.8–16.4)
Yes	435	5.6	39.8 (35.3–44.4)
Age of the mother			
< 20 years	402	5.1	8.2 (5.8–11.2)
20–30 years	4598	58.3	17.4 (16.4–18.6)
31–49 years	2883	36.6	17.7 (16.4–19.2)
Birth interval			
≤ 2 years	1017	15.7	16.00 (13.9–18.4)
2–3 years	2409	37.2	14.9 (13.5–16.3)
≥ 3 years	3048	47.1	18.8 (17.4–20.2)
Family size			
1–4	2267	28.8	18.4 (16.9–20.1)
5–9	5138	65.2	16.7 (15.7–17.74)
10+	478	6.1	14.8 (11.9–18.3)
Mass media exposure			
Have no exposure	3157	40.1	12.8 (11.6–14.06)
Have exposure	4708	59.9	20.0 (18.9–21.25)
Total	7883		17.08 (16.3–17.9)

*Any professional/technical/managerial, clerical, sales and services, skilled manual, unskilled manual and agriculture classifications were classified as working

Variables including wealth index, residence, maternal education, husband education, Community Conversation (CC), deworming tablet intake, antenatal care attendance, age, anemia status, birth interval, occupation, family size and mass media were found to have statistically significant associations with iron tablet intake at p-value <0.25, a cutoff point determined at bivariate analysis stage. Parity was not significantly associated at p-value 0.25 in the bivariate stage.

In the final multivariable model, awareness of Community Conversation (CC) program, deworming tablet intake, ANC attendance, mother’s age and family size were significantly associated with iron tablet intake during pregnancy. Those mothers who were aware of the CC program had 20% [AOR = 1.2; 95% CI: (1.01–1.40)] higher odds of taking iron tablets as compared with mothers who had no CC program awareness. The odds of taking iron tablets were 2.9 times (AOR = 2.9; 95% CI: (2.3–3.7) higher among those who took deworming tablets. Those mothers who attended the minimum four ANC visits as recommended by WHO were nearly 4 times [AOR = 3.9; 95% CI: (3.3–4.6)] more likely to take iron tablets as compared to those who did not attended the minimum recommended ANC visits. The odds of taking iron supplements during pregnancy were nearly 3 times [AOR = 2.9; 95% CI: (1.1–7.4)] higher among those mothers who were in the age group 31–49 years as compared to those mothers who were in the age group < 20 years. The odds of taking iron tablets during pregnancy were lower among those with larger family size. Those mothers with family size ≥ 10 had 32% [AOR = 0.68; 95% CI: (0.49–0.97)] lower odds of iron tablet intake during pregnancy (Table 2).

**Table 2:** Binary and multivariable logistic regression to identify factors associated with iron intake during pregnancy in Ethiopia, 2011

Variables	Crude Odd Ratio (COR 95%CI)	Adjusted Odd Ratio (AOR 95%CI)
Aware of CC program		
No	1.0	1.0
Yes	1.7 (1.5–2.0)	1.2 (1.04–1.4)**
Deworming tablet intake		
No	1.0	1.0
Yes	3.6 (2.9–4.4)	2.9 (2.3–3.7)*
ANC attendance		
< 4 times	1.0	1.0
≥ 4 times	4.5 (4.0–5.1)	3.9 (3.3–4.6)*
Maternal age		
< 20	1.0	1.0
20–30	2.4 (1.6–3.4)	2.5 (1.0–6.3)
31–49	2.4 (1.7–3.5)	2.9 (1.1–7.4)**
Family Size		
< 4	1.0	1.0
5–9	0.9 (0.8–1.0)	0.9 (0.8–1.1)
10+	0.8 (0.6–1.0)	0.7 (0.5–0.97)**

*p < 0.05, **p < 0.001

## Discussion

During pregnancy, the physiological requirement of iron is one of the public health difficulties to meet with most diets in developing countries [2–4, 20]. Pregnant women should routinely receive iron supplements in almost all contexts [21]. However, the coverage of iron supplementation during pregnancy remains low in Ethiopia. Only 17.1% of the pregnant mothers in this study took iron supplements. The geospatial analysis showed that Tigray, Addis Ababa and Harari regional states were statistically significant hot spot areas for iron supplementation during pregnancy. A possible justification could be because Addis Ababa and Harari are urban areas. Thus access to awareness of the iron supplementation program and its benefits might be better than in other geographic areas. The cold spot clusters are concentrated in Amhara region, particularly in the northwestern part of the region, central Oromiya, South nation, nationalities and people regional state of Ethiopia. The probable justification could be the antenatal coverage was low as compared to the hotspot areas [13]. ANC is the major modality to distribute iron tablet for pregnant mothers in Ethiopia. This study also found that those mothers who had attended the ANC had higher odds to receive iron tablet as compared to their counter parts. The national coverage of iron supplementation is lower as compared to available studies from Pakistan [22] and Tanzania [23].

A finding from a qualitative study done on health professionals in Southern Nation and Nationalities People state (SNNP) and Tigray regional states revealed that even though they are aware of the guideline, the practice in most health posts has been to distribute Iron folic acid (IFA) on a curative basis only to anemic women [24]. There was also poor understanding of the benefits of iron folic acid supplements for non-anemic pregnant women or their infants, particularly for seemingly healthy pregnant women. Additionally, there are some negative perceptions about iron and folic acid supplements (i.e. that they might make the baby bigger, or that they are bad for the baby) [24]. A study from North West Amhara in Ethiopia revealed that 28.5% of women believed that continuous uptake of iron folate supplementation leads to over-weight babies [25], which misleads mothers not to use iron supplements.

Having WHO minimum ANC attendance (at least four times) was associated with higher intake of iron tablets during pregnancy. Other studies have also reported that a higher number of ANC visits was associated with iron tablet use during pregnancy [22, 26]. In addition to the basic fact that more ANC visits means more interaction with a health provider, this could be due to the fact that when mothers attended ANC frequently, their hemoglobin level could be monitored continuously Pregnant women should routinely receive information on the signs of complications and be tested for them at all antenatal care visits. This helps the mother to receive iron tablet based on their hemoglobin progress.

Community Conversation (CC) is one of the programs implemented in rural communities of Ethiopia to communicate health information on different topics such as ANC, pregnancy and nutrition. In this study, those mothers who were aware of the CC program were more likely to receive iron tablets during pregnancy. The community conversation might help mothers to be aware of the advantages of taking iron tablets during pregnancy and ANC attendance. Women who were aware of the CC program would have a positive understanding of the benefits of taking iron tablets. A study from North West Amhara showed that those mothers who had good knowledge were more likely to be compliant with recommendation for iron tablet intake during their pregnancy compared to those who had low knowledge [25].

In this analysis, older maternal age was significantly associated with higher odds of taking iron tablets during pregnancy. This is consistent with a study from Tanzania, India and Sudan [23, 25, 27, 28]. The possible explanation could be that older mothers assume at greater risk for anemia due to repeated pregnancy and supplemented iron for preventing anemia. Older women may also be more concerned about their health and pregnancy outcomes, receive necessary support and cooperation from their family members and have had more better experience in prevention and treatment of iron deficiency anemia [25]. Public health messages may not have effectively reached younger age groups.

In this study, pregnant mothers who took deworming tablets had higher odds of taking iron tablets during pregnancy. In populations with endemic hookworm, anti-helminthic therapy should be given presumptively to anyone with severe anemia, because treatment is safe and much less expensive than diagnosing hookworm infection [21, 28]. Interaction with health workers for deworming tablets would increase pregnant women’s uptake of recommended iron tablets. Those mothers from households with a larger family size (≥10) were less likely to receive iron tablets during pregnancy. This could be due to the fact that those mothers might attend less ANC due to higher burdens of responsibility in their households to care for multiple family members including children.

This study has limitations. This is a secondary data analysis which missed key variables that potentially determine iron supplementation use during pregnancy in a wider perspective. Potential explanatory variables such as availability of iron tablet in the health institution and knowledge of pregnant mothers were not assessed. Some regions had small sample size, which questions the accuracy of coverage estimates per region, so that it should be interpreted in caution.

## Conclusion

Differences in iron supplementation use during pregnancy was observed in Ethiopia with relatively higher coverage of iron supplementation areas found in Northern eastern Tigray, central Ethiopia near Addis Ababa and Eastern parts of Ethiopia near Dire Dawa and Harari. Attention should be given to mothers of younger age and those with large family size. Promotion of ANC services based on the WHO standard can be used as an intervention for improving iron supplementation during pregnancy.

## Abbreviations

ANC: Antenatal care
AOR: Adjusted odd ratio
CC: Community conversation
CDC: Centers for Disease Control and Prevention
EDHS: Ethiopian Demographic and Health Survey
GIS: Geographic Information System
IFA: Iron folic acid
SNNP: Southern Nations Nationalities and Peoples
VIF: Variance inflation factors
WHO: World Health Organization
